# Habitat partitioning among sympatric tinamous in semiarid woodlands of central Argentina

**DOI:** 10.1371/journal.pone.0297053

**Published:** 2024-01-19

**Authors:** Eduardo T. Mezquida, Juan I. Zanón-Martínez

**Affiliations:** 1 Department of Ecology, Faculty of Sciences, Autonomous University of Madrid, Madrid, Spain; 2 Biodiversity and Global Change Research Center (CIBC-UAM), Autonomous University of Madrid, Madrid, Spain; 3 Centro para el Estudio y Conservación de las Aves Rapaces en Argentina, Universidad Nacional de La Pampa, Santa Rosa, La Pampa, Argentina; 4 Instituto Multidisciplinario sobre Ecosistemas y Desarrollo Sustentable, CONICET, Universidad Nacional del Centro de la Provincia de Buenos Aires, Tandil, Buenos Aires, Argentina; UFERSA: Universidade Federal Rural do Semi-Arido, BRAZIL

## Abstract

Sympatric, phylogenetically related and morphologically similar species that overlap in their distributions at a regional scale display different patterns of co-occurrence at local assemblages. Occurrence of each species at local scales might be the result of interspecific competition for limiting resources. However, these patterns could also arise from species-specific habitat preferences along the abiotic or land use gradients. To assess the role of these mechanisms, we investigated niche partitioning among sympatric tinamou species occurring in semiarid woodlands of central Argentina. We used occupancy models incorporating habitat characteristics and interspecific interactions, while accounting for detectability, to examine the spatial overlap among elegant crested tinamous (*Eudromia elegans*), brushland tinamous (*Nothoprocta cinerascens*), and nothura tinamous (*Nothura darwinii* and *N*. *maculosa*) across a wide regional scale. In addition, we investigated time partitioning among these species by estimating the degree of overlap in their daily activity patterns. The regional distribution of the three species was influenced by the gradient in plant productivity and vegetation structure, in agreement with their broad habitat requirements. We also found that the occurrence of each species was presumably affected by the presence of one or two predator species. Models including interactions among tinamou species found weak negative and positive interactions among species pairs, suggesting that co-occurrence patterns were mainly driven by species-specific habitat use rather than interspecific competition. The three species were diurnal, showing two main peaks of activity, and overlapped widely in their overall diel activity, although subordinate species tended to shift their activity patterns to reduce encounters with the dominant tinamou species, suggesting some segregation in this niche dimension. Projected changes in rainfall seasonality and warmer conditions in this region could benefit elegant crested tinamous over the other two species, although climate and land use changes will likely have a negative impact on all tinamou species.

## Introduction

Species distributions are shaped by the interaction between abiotic, biotic and historical factors [1–3]. Abiotic factors determine the suitable ecological space where species can potentially occur [4, 5]. However, species do not usually occupy all suitable areas due to factors such as colonization history, dispersal ability, geographical barriers, and contemporary human impacts [2, 6, 7]. Moreover, complex interactions such as the presence of other species that may act as competitors, predators, or pathogens, further reduce the geographical ranges of species [4, 8]. Food availability could also limit the occupancy of suitable habitats and increase competition among co-occurring ecologically similar species [9, 10].

Sympatric similar species that overlap in their distributions at a regional scale display different patterns of co-occurrence at local assemblages [11–13]. Co-occurrence patterns in these ecologically or closely related species might be the result of interspecific competition [14]. Therefore, the presence or abundance of a species that is a superior competitor can restrict the distribution of the inferior competitor, or even completely exclude it, when resources shared by both species are limiting [15, 16]. Niche theory predicts that co-occurring competing species should partition their niches to achieve stable coexistence [17]. This partition or segregation could involve one or several niche dimensions (e.g., spatial, temporal, diet) [18–20]. For example, species overlapping widely in their distribution could reduce interspecific competition by partitioning the spatial niche at lower scales, such us microhabitat, showing low overlap in their activity patterns to limit behavioral interactions, or using different foraging strategies [19–21].

Conversely, observed patterns of co-occurrence among species could arise from their different habitat preferences [22, 23]. Hence, habitat characteristics along abiotic or land use gradients would be the main determinant of species distribution and co-occurrence at local scales [23, 24]. Nevertheless, other mechanisms can explain co-occurrence patterns of similar species, or act concurrently to shape species coexistence [25–28]. For example, the presence or abundance of predators shared by prey species could affect the coexistence of prey species depending on their vulnerability or behavioral responses to predators [29, 30]. Moreover, human activities can alter the occurrence patterns of interacting species by, for example, increasing hunting pressure on target species, or indirectly through habitat modification [31–33].

Disentangling the role of competition and habitat requirements from patterns of species co-occurrence needs to deal with imperfect detection during sampling [12, 14, 24]. For example, species absence at some sites could result from non-detection rather than true absences, leading to ambiguous interpretations of co-occurrence patterns [34, 35]. Occupancy models allow for the estimation of occupancy for each species while accounting for imperfect detection, and to examine whether the occurrence of one species is affected by the presence of a potential competitor [12, 36]. In addition, other explanatory variables, such as habitat characteristics, predator occurrence, or land use, can be included as covariates in the detection or occupancy part of the models to determine their influence on species distributions and co-occurrence patterns [34, 37].

In this study, we explore the mechanisms of niche partitioning in sympatric tinamou species occurring in semiarid woodlands of central Argentina. We specifically investigated the overlap in spatial and temporal activity patterns among tinamou species across a wide regional scale to encompass various landscapes as a result of abiotic gradients and human uses. Tinamous (Aves: Tinamidae) are a family of Neotropical birds found in Central and South America, that comprise 46 ground-dwelling species, with similar body type [38]. They are medium-sized birds with stout bodies that taper towards the tail, short and rounded wings, and legs with long toes for walking on uneven terrain [39]. Tinamous occur in various environments, from cloud forests, to desert scrub and grassy steppes, where they usually defend small territories [40–42]. Tinamous’ diet consist mostly of invertebrates, leaves, fruits, and seeds, although species differ in the relative use of each resource type in their diet [39, 43]. Therefore, their similar morphology and ecological requirements suggest that competition could be a potential mechanism of niche segregation between co-occurring tinamou species.

Four tinamou species occur in semiarid woodlands of central Argentina: elegant crested tinamous (*Eudromia elegans*), brushland tinamous (*Nothoprocta cinerascens*), Darwin’s nothuras (*Nothura darwinii*), and spotted nothuras (*Nothura maculosa*). Darwin’s and spotted nothuras are similar species, and earlier authors considered Darwin’s nothuras to be subspecies of spotted nothuras [44]. Given the similarity between both species and the natural variation in plumage coloration, we could not discriminate between both species with our recording methodology (see Methods), so we denote both *Nothura* species as nothura tinamous hereafter. The semiarid woodlands of central Argentina are dominated by caldén (*Neltuma caldenia*; formerly *Prosopis caldenia*), a tree species endemic to the southernmost part of the Espinal phytogeographic province [45], so these woodlands are singular natural habitats of conservation value. In addition to the abiotic gradients, this savanna-like ecosystem has been historically transformed and impacted by different land uses, creating a variety of landscapes [46, 47].

Here, we examined the spatial distribution of each tinamou species and its relationship to habitat characteristics and predator assemblages in these human-modified woodlands to address the hypothesis that species-specific habitat use is the main determinant of these species occurrence patterns at a regional scale. Then, we test whether interspecific competition might explain the spatial co-occurrence of species after accounting for their habitat use. Finally, we estimated the temporal activity patterns of each species and their overlap to test for shifts in activity patterns as a potential mechanism to lower behavioral interactions.

## Methods

### Study area

**Fig 1:**
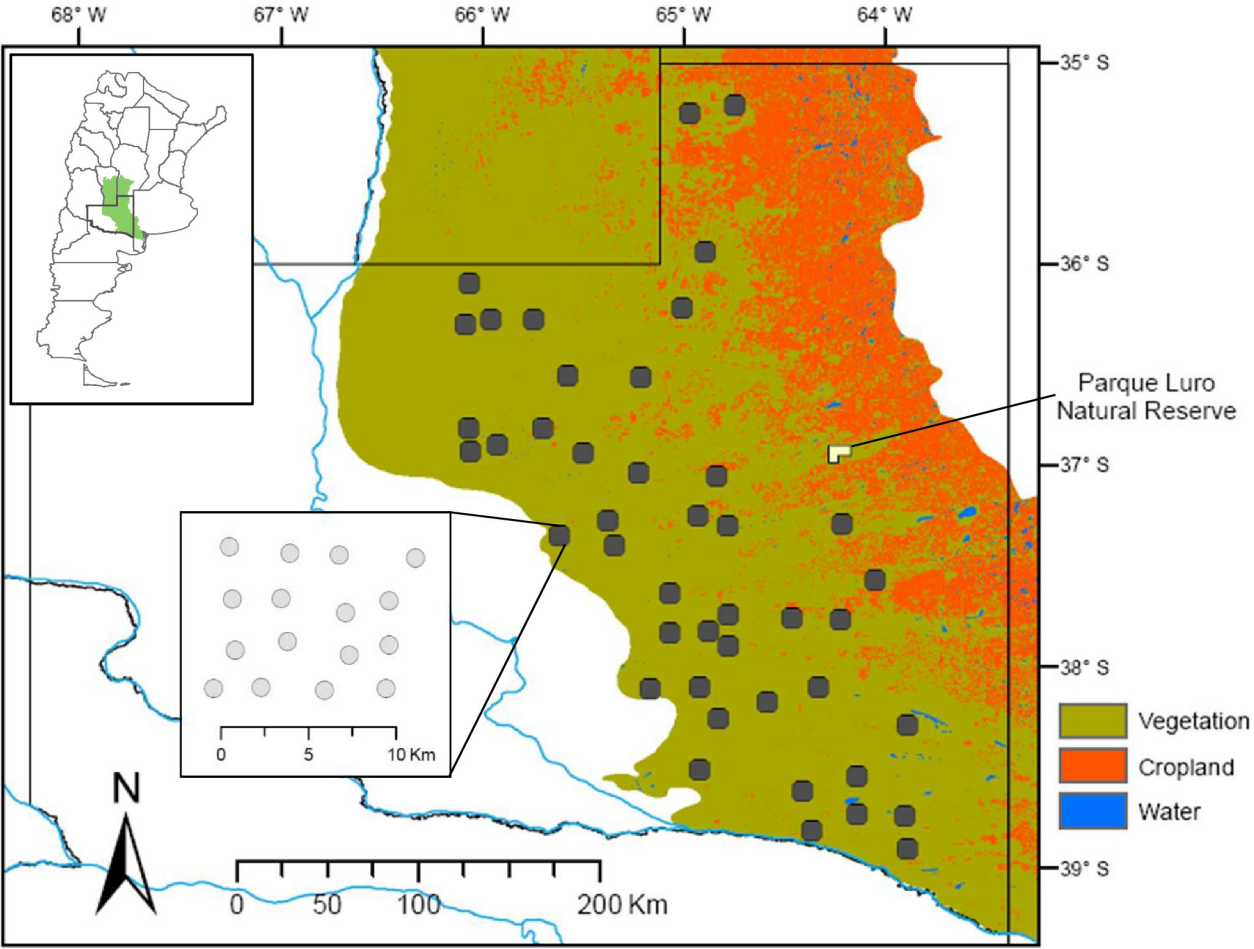
Map of the study area in the caldén woodland region represented by green in the upper inset, within the political boundaries of La Pampa province, Argentina, and the 45 (10 × 10 km) grid square locations selected in current caldén woodlands marked with black dots. The central inset shows an example of the sixteen camera traps installed at regular intervals in a 4 × 4 array within the grid. Background map: political boundaries and rivers were downloaded from data provided by Instituto Geográfico Nacional de la República Argentina (https://www.ign.gob.ar/NuestrasActividades/InformacionGeoespacial/CapasSIG), and the caldén woodland region was obtained from Ministerio de Medioambiente y Desarrollo Sostenible de la República Argentina (https://www.argentina.gob.ar/ambiente/bosques/primer-inventario-nacional-bosques-nativos). Land cover data was downloaded from data provided by the University of Maryland (https://glad.earthengine.app/view/global-land-cover-land-use-v1) under CCBY 4.0 International License.

We conducted the study in the caldén woodland region in La Pampa province, central Argentina, comprising about 80,000 km^2^ (Fig 1). These semiarid woodlands are dominated by caldén trees, with tree cover ranging from 30 to 50%, and an understory where perennial grasses predominate over shrubs. The topography is relatively flat and characterized by plateaus, valleys, hills, and low-altitude plains (< 200 m). The climate is continental semiarid with 15°C of annual average temperature and 550 mm of annual precipitation concentrated during the spring and summer months. Most of the land in this region is privately owned and managed for livestock (mainly cattle), sport hunting, and, to a lesser extent, cultivation; only < 1% of the caldén woodland is protected in a local reserve (Parque Luro Natural Reserve; Fig 1) [46].

### Data collection

#### Camera trap survey

We used passive camera traps to record the presence of tinamou species, a valid method to sample ground-dwelling birds [24, 48, 49]. We divided the study area (i.e., the actual distribution of caldén woodlands) into a grid of 10×10 km cells from which we randomly selected 45 of them (~8% of the total area). We installed 16 single, remote cameras (Digital Moultrie Game Spy 4.0 Camera, EBSCO Industries) at regular intervals (2–3 km) in a 4×4 array within each selected 10×10 km square (Fig 1) between September 2010 and March 2013, for a total of 720 sites. Although the distance between cameras was relatively long for our study organisms, this sampling design allowed us to record large carnivores and better estimate the occurrence of potential tinamou predators [50]. We installed and georeferenced the cameras along dirt roads, wildlife and cattle trails, following standard practices [20, 24, 51], operating four grids simultaneously. The cameras functioned 24 h per day for an average of 31 days, taking a photograph with a minimum 5-min delay between triggering events. We did not use a shorter delay (e.g., 15–30 s) as in other studies due to the large number of cattle in the area that might cause memory cards to fill [50].

We recorded the date, time, camera ID, and species for each photograph taken during the study period to create capture histories for each tinamou species. We used daily capture events and recorded the detection or non-detection of each tinamou species to build capture histories at each of the remote camera locations. To describe temporal daily activity patterns, we considered photos of each tinamou species as a single capture event if they occurred within 30 min of a previous photo [20].

#### Environmental variables

We considered a set of climatic, habitat, anthropogenic, and biotic variables expected to influence the spatial distribution of tinamous. We used monthly climate data for our studied period (2010–2013) from the CHELSA dataset [52] at 30-s resolution and calculated eight bioclimatic variables using the *terra* package [53] in R 3.6.1 (R Core Team). The eight bioclimatic variables represent annual trends (mean annual temperature and annual precipitation), seasonality (annual range in temperature and precipitation), and extreme or limiting environmental factors (temperature and precipitation of the wet and dry quarters). We used climate engine (https://climateengine.org) to obtain the mean value of the Enhanced Vegetation Index (EVI) for the year 2010 derived from Landsat at 30-m spatial resolution. This index quantifies vegetation greenness and was used as a proxy of the spatial variation in plant productivity and biomass [54]. We acquired data on habitat from the National Forest Administration of Argentina, which used remotely sensed data and extensive field surveys [47]. Using a vector version of the map, we recoded land-cover classes as closed caldén woodlands (closed woodlands and more open woodlands with shrubs), open caldén woodlands (open and savanna-like woodlands), shrublands, and grasslands. Climatic and habitat variables were obtained within a 200-m radius buffer around each camera station. Bioclimatic variables and EVI were estimated as the mean value and habitat variables as the proportion of each land-cover class within the buffer (12.6 ha). This spatial scale is representative of tinamous’ home ranges or core areas (e.g., 16–19 ha for spotted nothuras, and 24 ha for brushland tinamous [41, 42]).

We used camera-trap data to derive anthropogenic and biotic variables. We calculated a relative activity level (or encounter rate) of cattle for each camera station to account for the potential influence of ranch practices. We also estimated the spatial variation in activity levels for potential predators of tinamous and their eggs, including puma (*Puma concolor*), pampas fox (*Lycalopex gymnocercus*), Geoffroy’s cat (*Leopardus geoffroyi*), pampas cat (*Leopardus colocolo*), Molina’s hog-nosed skunk (*Conepatus chinga*), and armadillos (*Chaetophractus villosus* and *Zaedyus pichiy*) [44, 55–57]. The encounter rates for cattle and each predator were calculated as the number of photographic events per day obtained per camera divided by the number of days cameras were operative.

We considered another set of variables hypothesized to affect tinamous’ probability of being photographed. Tinamous are mostly resident and non-migratory, although movements and social behavior may change between seasons. For example, elegant crested tinamous frequently form flocks during the non-breeding season [58, 59]. Thus, we estimated two temporal variables: Julian date; the date when each camera started to operate (day 1 = 1 October), and season; a dichotomous variable representing austral spring-summer (October-March) or autumn-winter (April-September). We characterized the habitat surrounding each camera site using land-cover classes from the National Forest Administration of Argentina (i.e., closed and open caldén woodlands, shrublands, and grasslands), as explained above, included a dichotomous variable indicating whether the camera was installed in a trail or not, and added trapping effort for each camera station (i.e., the number of days each camera was active) [60]. We also included the occurrence of humans (i.e., encounter rates derived from camera-trap records) to account for the potential effects of human interference on detection probability of tinamous.

### Data analysis

#### Single species occupancy models

We used single species occupancy models to assess the spatial distribution and overlap of tinamou species. Occupancy models estimate the probability of a site being occupied by a species while accounting for imperfect detection (i.e., detection probability derived from detection-nondetection information; [35, 37]). We modeled the occurrence of each tinamou species, coding a 1 if a tinamou species was detected at site *i* and a 0 if a tinamou was not detected at site *i*, as a Bernoulli random variable, *Zi* ~ Bernoulli (ψ*i*), where ψ*i* is the probability that a tinamou species occurred at camera station *i* while correcting for imperfect species detection data [35, 37]. In the case where the species is not detected, occupancy state is ambiguous, either the site was occupied, but the species was not detected (i.e. species present but not photographed), or the site was unoccupied [35, 37]. Daily photographic events, *yi*,*j* (or detection/non-detection histories *yi*,*j*), are another Bernoulli random variable with a success rate that is the product of the actual occurrence at site *i*, *Zi*, and detection probability *p* at site *i* during survey *j* (i.e. trap day *j*), *yi*,*j* | *Zi* ~ Bernouilli (*pi*,*j* * *Zi*), expressed as conditional on actual occurrence [35, 37]. We used a set of environmental covariates expected to influence the probability of occupancy and detection of each tinamou species. We assumed that habitat characteristics did not change during the study period, mostly because there were not fires in the study sites (last wildfires occurred an average of 11 [± 1 SD] years before starting the camera-trapping surveys; https://firms.modaps.eosdis.nasa.gov) and vegetation structure remains relatively constant in these semiarid woodlands in the absence of fire disturbances. We built occupancy models for each tinamou species in the package *unmarked* [61]. We first examined correlation coefficients between our candidate covariates to avoid multicollinearity. We removed six bioclimatic and one land-cover variables because of their high correlation coefficients, and used 17 variables (standardized to zero mean and unit variance) as predictors in the models (variance inflation factor values < 2.6; in all cases) (S1 Table).

We followed a two-step approach to build occupancy models for each tinamou species. First, we evaluated the effect of covariates on detection probability while holding occupancy constant. Thus, we fitted models including closed woodland, open woodland, shrubland, human activity, camera stations installed in trails, and trapping effort as predictors in the detection part of the models. We also included season in the detection models for elegant crested and brushland tinamous, or Julian date (and its quadratic term) in the models for nothura tinamous (they were solely recorded during spring-summer). Then, we used the best detection model for each species to explore the effects of covariates on occupancy. Predictors of occupancy models included temperature and precipitation seasonality, EVI, closed woodland, open woodland, shrubland, and encounter rates of cattle, puma, pampas fox, Geoffroy’s and pampas cats, Molina’s hog-nosed skunk (skunk; hereafter), and armadillos. We also included the interactions between temperature or precipitation seasonality and EVI. We ranked our candidate models according to the Akaike’s Information Criterion (AIC; [62]) and reported top-ranked models (i.e., those within ΔAIC ≤ 2 of the top model).

#### Multispecies occupancy models

We fitted multispecies occupancy models to investigate the potential spatial interactions between tinamou species [63]. We used the best detection and occupancy covariates from the single species occupancy models to build the individual species parts of the multispecies occupancy model with the three tinamou species (note that we refer to three species because the two *Nothura* species were considered as one taxon). We modeled the co-occurrence of each pair of species and the co-occurrence of the three tinamou species as constant in all cases (i.e, without including covariates potentially affecting pair-wise interactions or the interaction among the three species), to minimize model complexity [32]. We evaluated and ranked multispecies occupancy models according to AIC in the package *unmarked*.

#### Time activity and overlap

We used time recordings from each camera trap to describe the daily activity pattern of each tinamou species and examine their temporal co-occurrence. To examine whether tinamou species shifted their daily activity patterns when co-occurring with another potentially dominant tinamou species, we split the data according to the median of the distribution of values of occupancy (habitat use) probability from the top single species occupancy model for the dominant species. We assumed that larger tinamou species are dominant to smaller ones, so we considered elegant crested tinamous (676–749 g; on average) as dominant over the other two tinamou species, and brushland tinamous (479–573 g) as dominant over nothura tinamous (214–274 g) [44, 55–57]. We fitted kernel density functions to time of tinamou detections and calculated the coefficient of overlap (Δ_1_) between each pair of species using the *overlap* package [64]. The coefficient of overlap calculates the area lying under two density curves, ranging from 0 (no overlap in activity patterns) to 1 (identical activity patterns). Confidence intervals for overlap coefficients were estimated from 10,000 bootstrap samples [64].

#### Ethics statement

Natural Resources Agency of La Pampa Province provided permission to conduct this research (no permit number), and property owners provided permission to access their lands and conduct the surveys.

## Results

### Camera-trapping survey

Fourteen of the 720 camera traps malfunctioned and were excluded from the analyses. Thus, total trapping effort was 35,300 trap-days across the 706 camera-trap locations (mean ± SD = 30.7 ± 7.5 trap days per camera station). We recorded 840 tinamou independent events, including 455 elegant crested, 270 brushland and 115 nothura tinamous. Elegant crested tinamous were recorded in 174 of the 706 study locations (i.e., a naïve occupancy of 25%), brushland tinamous in 114 (16%), and nothura tinamous in 57 (8%).

### Spatial distribution

Daily detection probability was highest for elegant crested tinamous (0.075 ± 0.029), followed by brushland tinamous (0.053 ± 0.024) and nothura tinamous (0.029 ± 0.026). Several covariates affected detection probabilities of each tinamou species (S2 and S3 Tables). Detection of elegant crested tinamous decreased in shrublands, with higher human activity, with more trapping effort (likely due to more survey effort was made in sites less used by this species), and increased in sites with cameras installed in trails (S2 Table). Detection also tended to increase during autumn-winter and decrease in closed woodlands (although 95% confidence intervals overlapped 0 in both cases; S2 and S3 Tables). Brushland tinamou detection decreased during autumn-winter and increased in shrublands and with cameras installed in trails (S2 Table). In addition, sites in closed forests and with more trapping effort tended to have higher detection probabilities (S2 and S3 Tables). Nothura tinamous were detected only during spring and summer, but their detections peaked in spring and declined into summer, and increased with more trapping effort (S2 Table). Detection in this species also tended to decrease in open woodlands and with increasing human activity (S2 and S3 Tables).

**Fig 2:**
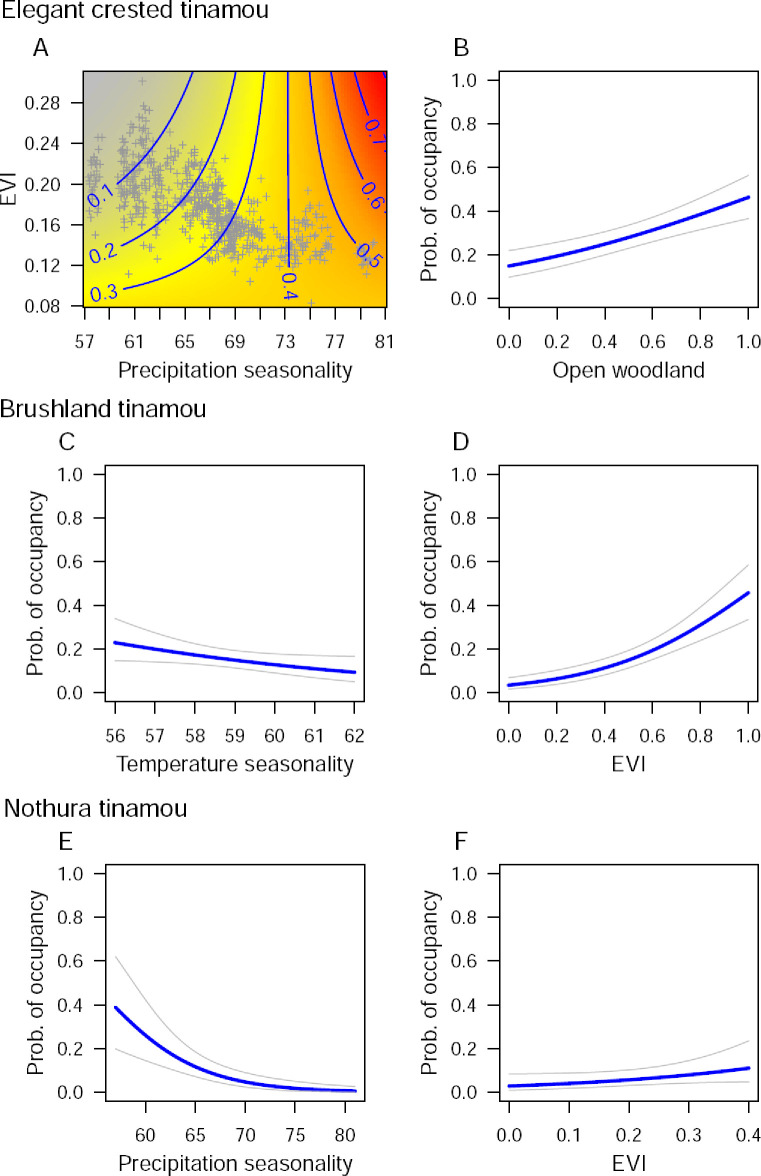
The interaction between precipitation seasonality and EVI, and its effect on the probability of occupancy (continuous response surface plot) (A); and relationships between the proportion of open woodland (B), temperature seasonality (C), precipitation seasonality (E), and EVI (D and F), and the probability of occupancy, as estimated by the most supported single-species occupancy models for three tinamou species. Grey plus signs denote the observed covariate values across the 706 study locations in caldén woodlands of central Argentina. Grey lines show the 95% confidence intervals.

After controlling for detection probability, elegant crested tinamous occupied 0.29 ± 0.01 (SE) of the study area (S1 Fig). Brushland tinamous were less widespread (0.21 ± 0.01), and nothura tinamou occupancy was more limited (0.14 ± 0.01; S1 Fig). Seasonality variables (mainly precipitation) and EVI influenced occupancy probability of the three tinamou species (Table 1 and Fig 2). The predicted occupancy of elegant crested tinamous increased with higher precipitation seasonality and lower EVI (Table 1), especially where seasonality was high and biomass low (Fig 2A; significant interaction between precipitation seasonality and EVI). Brushland tinamou predicted occupancy was negatively associated with temperature seasonality (Table 1 and Fig 2C), and positively with EVI (Table 1 and Fig 2D), whereas nothura tinamou occupancy decreased with higher precipitation seasonality (Table 1 and Fig 2E), and increased with higher EVI (Table 1 and Fig 2F). Occupancy of elegant crested tinamous also increased in open caldén woodlands (Fig 2B) and shrublands, and correlated positively with cattle encounter rates (Table 1 and S3 Table). Finally, occupancy of the three tinamou species was related to the encounter rates of potential predators (Table 1 and S3 Table). Occupancy of elegant crested tinamous decreased with higher encounter rates of pampas cats, and those of Brushland and nothura tinamous were positively associated with Geoffroy’s cat encounter rates (Table 1 and S3 Table). Brushland tinamou occupancy was also positively correlated with skunk encounter rates (Table 1 and S3 Table).

**Table 1:** Top single-species occupancy models (ΔAIC ≤ 2) used to evaluate the effects of climate, habitat, anthropic and biotic variables on the probability of occupancy (*Ψ*) and detection (*p*) of three sympatric tinamous in the caldén woodland region in central Argentina. Variables with greater effect (i.e., 95% confidence intervals do not include zero) are marked in bold. Positive (+) and negative (-) signs denote direction of explanatory variables. Variable names refer to temperature seasonality (Tseas), precipitation seasonality (ppseas), enhanced vegetation index (EVI), closed caldén woodlands (closed woodland), open caldén woodlands (open woodland), shrublands (shrubland), and encounter rates of cattle (cattle), Geoffroy’s cat (gcat) and pampas cat (pcat). Covariates influencing the probability of detection correspond to the best model fitted for each tinamou species (see S2 Table); *p*(general1): *p*(+autumn-winter, -closed woodland, -shrubland, -human activity, +trail, -trapping effort); *p*(general2): *p*(-autumn-winter, +closed woodland, +shrubland, +trail, +trapping effort); *p*(general3): *p*(-date, -date^2^, -open woodland, -human activity, +trapping effort).

Species/Model	AIC	ΔAIC	*W*	*K*
Elegant crested tinamou				
*Ψ* (**+ppseas**, **-**EVI, **+ppseas×EVI**, **+open woodland**, +shrubland, +cattle, **-pcat**); *p*(general1)	3731.45	0.00	0.28	15
*Ψ* (**+ppseas**, **-**EVI, **+ppseas×EVI**, **+open woodland**, **-pcat**); *p*(general1)	3732.00	0.54	0.21	13
*Ψ* (**+ppseas**, **-**EVI, **+ppseas×EVI**, **+open woodland**, +shrubland, +gcat, **-pcat**); *p*(general1)	3732.28	0.83	0.19	15
*Ψ* (**+ppseas**, **-**EVI, **+ppseas×EVI**, **+open woodland**, +cattle, **-pcat**); p(general1)	3733.04	1.59	0.13	14
Brushland tinamou				
*Ψ* (**-Tseas**, **+EVI**, **+gcat**, **+skunk**); *p*(general2)	2398.84	0.00	0.38	11
*Ψ* (**-Tseas**, **+EVI**, +closed woodland, **+gcat**, **+skunk**); *p*(general2)	2399.88	1.05	0.23	12
*Ψ* (**-Tseas**, **+EVI**, +Tseas×EVI, **+skunk**); *p*(general2)	2400.02	1.18	0.21	11
Nothura tinamou				
*Ψ* (**-ppseas**, **+**EVI, **+gcat**); *p*(general3)	1133.91	0.00	0.38	11
*Ψ* (**-ppseas**, +**gcat**); *p*(general3)	1135.02	1.11	0.22	9
*Ψ* (**+**Tseas, **-ppseas**, **+**EVI, +**gcat**); *p*(general3)	1135.74	1.83	0.15	11

#### Co-occurrence between species

**Fig 3:**
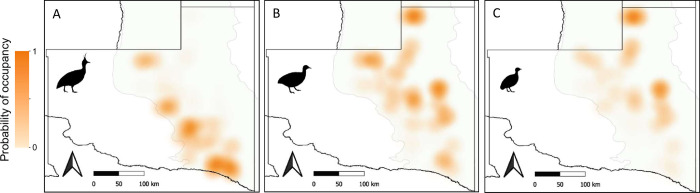
Probability of occupancy (site use) estimated for elegant crested tinamous (A), brushland tinamous (B), and nothura tinamous (C) in the caldén woodland region of central Argentina. Occupancy probability for each tinamou species was estimated from single-species occupancy models, and values for the 706 study locations are represented as a heatmap using QGIS (QGIS Development Team). Political boundaries were downloaded from data provided by Instituto Geográfico Nacional de la República Argentina (https://www.ign.gob.ar/NuestrasActividades/InformacionGeoespacial/CapasSIG).

**Fig 4:**
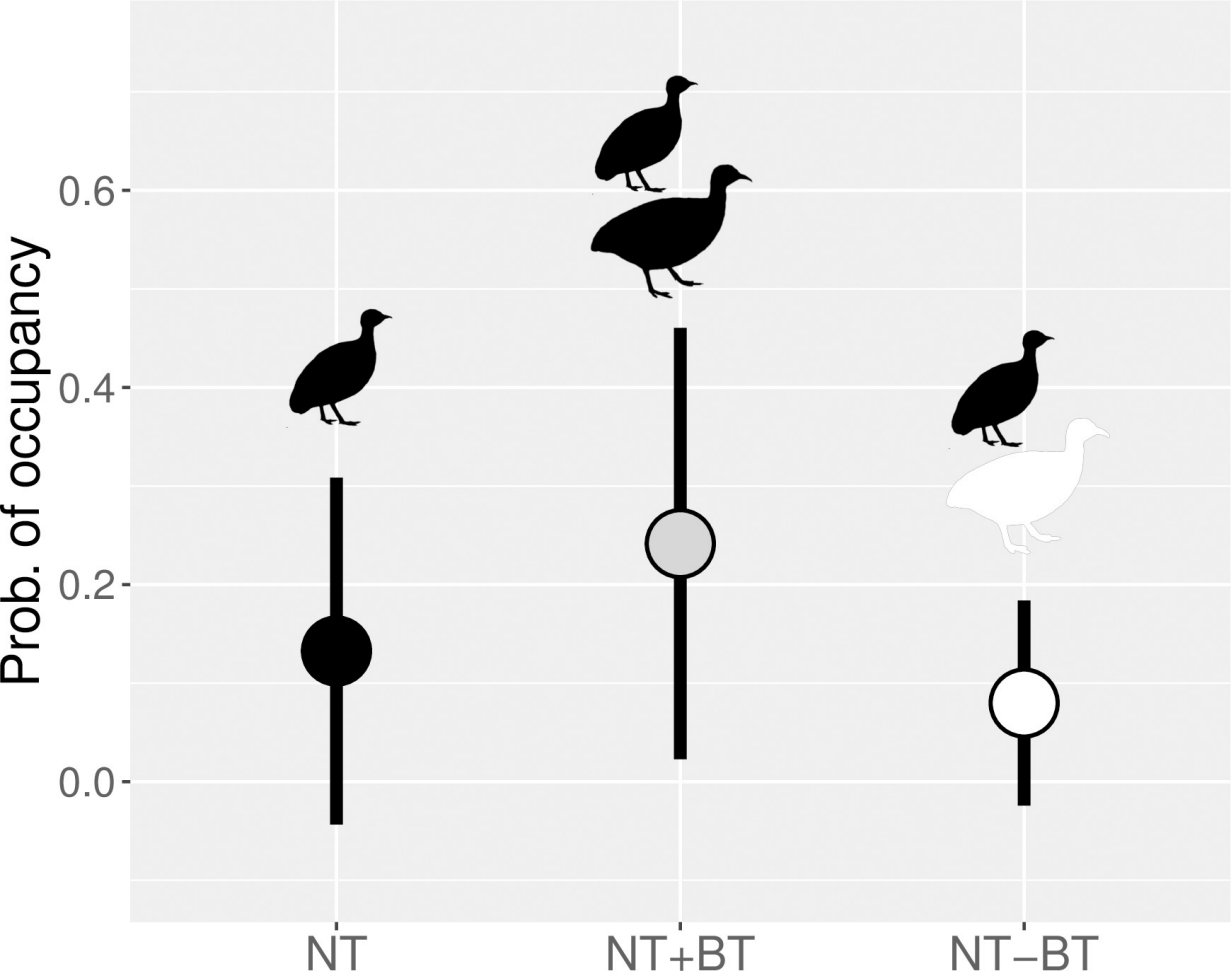
Probability of occupancy (± SD) for nothura tinamous (NT; black dot) in caldén woodlands of central Argentina, in areas where brushland tinamous are present (NT+BT; grey dot) and absent (NT-BT; white dot); as estimated by the most parsimonious multispecies occupancy model.

The most supported multispecies occupancy model included a positive interaction between brushland and nothura tinamous (Table 2). The next model included a weak negative interaction between elegant crested and nothura tinamous (i.e., confidence interval overlapping 0; Table 2); although this model was less well supported by the data (Table 2). Elegant crested tinamous mainly occupied the western portion of the study area (Fig 3A), whereas nothura tinamous were more likely found in the eastern portion (Fig 3C). Actually, spatial co-occurrence between elegant crested and nothura tinamous was low (mean predicted overlap: 0.014 ± 0.001 SE). Although nothura tinamou was the less widespread species, spatial co-occurrence between brushland and nothura tinamous was higher (0.087 ± 0.006) than it was between elegant crested and nothura tinamous (Fig 3), and areas where brushland tinamous were present were more likely occupied by nothura tinamous (Fig 4).

**Table 2:** Multi-species occupancy models using the detection and occupancy covariates from the best single-species models fitted for elegant crested, brushland and nothura tinamous (see S2 Table, Table 1; for the detection and occupancy parts of the model for each tinamou species), including interactions between each pair of species and the three-species interaction. Species interactions were modeled as constant in all cases (i.e., *Ψ*(.)). Beta estimates and *P*-values are shown for the interaction between species included in each model.

Model (species interactions)	AIC	ΔAIC	*W*	*K*	Estimate	*P*
brushland:nothura(.)	7254.00	0.00	0.64	37	1.641	0.001
elegant crested:nothura(.);	7255.99	2.00	0.24	38	-0.029	0.959
brushland:nothura(.)					1.641	0.001
elegant crested:brushland(.);	7257.88	3.88	0.09	39	0.125	0.739
elegant crested:nothura(.);					-0.092	0.879
brushland:nothura(.)					1.651	0.001
elegant crested:brushland(.);	7259.80	5.80	0.04	40	0.176	0.409
elegant crested:nothura(.);					0.064	0.784
brushland:nothura(.);					1.703	0.530
elegant crested:brushland:nothura(.)					-0.350	1.200

#### Activity patterns and overlap

**Fig 5:**
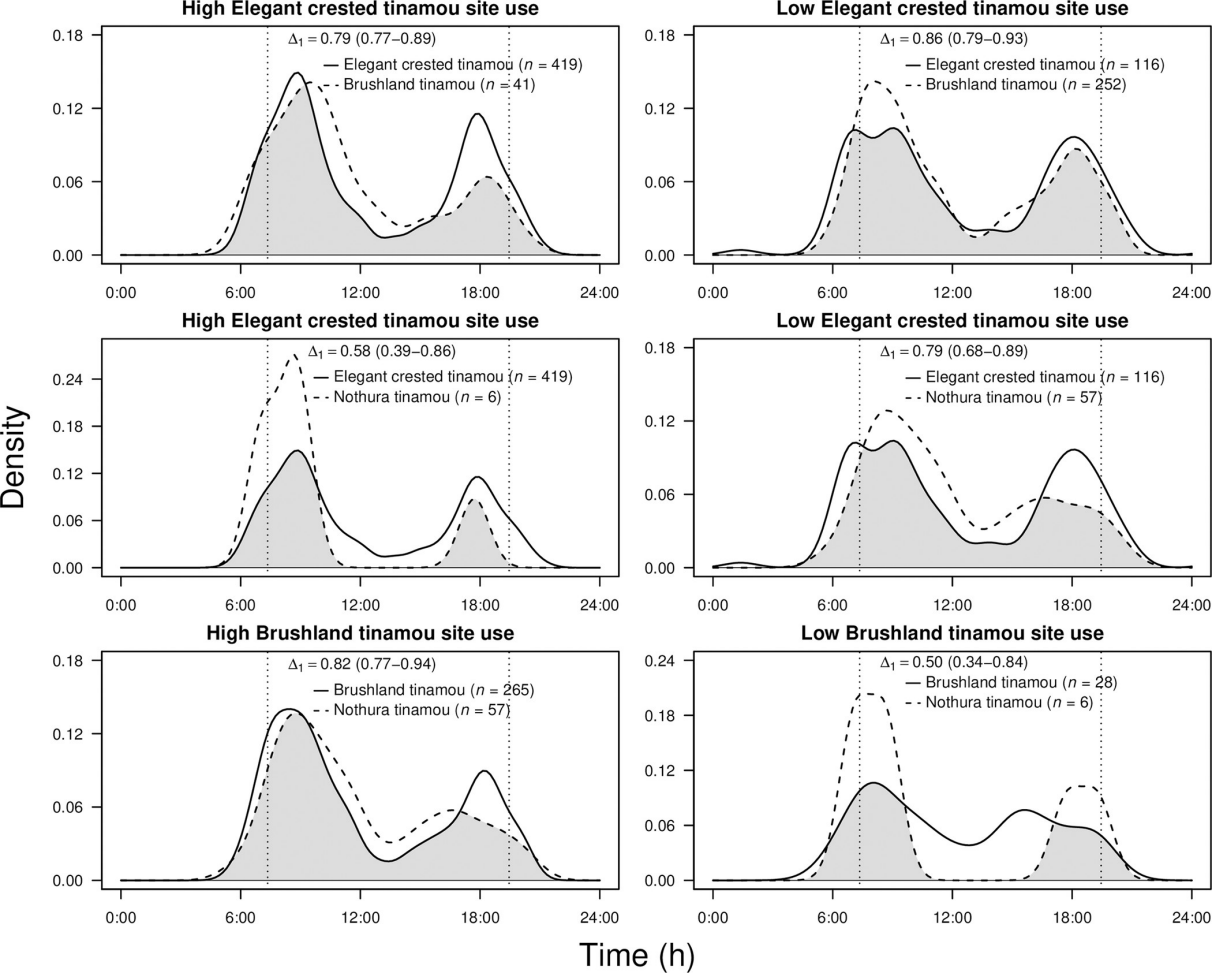
Kernel density estimation on circular data from camera-trapping records of activity patterns of three tinamou species in areas of high and low occupancy (site use) of the potentially dominant tinamou species in caldén woodlands of central Argentina. Grey shading indicates the overlap in species activity and is reported as the coefficient of overlap (Δ_1_) with 95% confidence intervals between brackets. Sample sizes in parentheses indicate the number of detections for each species. Vertical black dashed lines represent average sunrise and sunset at the study area.

We used a total of 891 tinamou detections to describe the activity pattern of each species. The three tinamou species exhibited diurnal activity with two main peaks, one during early morning and another during late afternoon (S2 Fig). This second peak of activity was less pronounced in nothura tinamous, due to this peak was more conspicuous during autumn-winter and this species was only detected during spring and summer (S3 Fig). Overall, the three species showed a significant overlap in their activity patterns (range: 0.84–0.91; S2 Fig). When splitting the data into high and low occupancy (habitat use) of the potentially dominant species, the overlap in activity patterns between elegant crested tinamous and the other two tinamou species was lower in sites of high elegant crested tinamou occupancy than in those of low habitat use by this species (Fig 5). However, the overlap in activity patterns between brushland tinamous and nothura tinamous was higher in sites of high brushland tinamou occupancy compared to those less used by this species, although sample size was low for some habitat use categories (Fig 5).

## Discussion

Our study combined large-scale camera-trap data, covering a gradient of environmental conditions and human impacts derived from varied land uses, and occupancy models to assess the relative role of environmental characteristics and interspecific interactions on the spatial distribution and co-occurrence patterns in sympatric tinamou species. Our results indicate that species-specific habitat partitioning was the main driver of tinamou distributions in semiarid caldén woodlands of central Argentina. We also found evidence suggesting positive as well as weak negative interactions between pairs of co-occurring species after accounting for detectability and habitat use. Moreover, our results suggest temporal partition in daily activity patterns where subordinate tinamou species co-occur with the potentially dominant elegant crested tinamou.

### Habitat partitioning

Our occupancy models included temporal, habitat, anthropic, and survey covariates, which had an effect on the detection probability of each tinamou species. After accounting for detectability, spatial occurrence of each species in these woodlands were consistent with its broad habitat requirements. At a geographical scale, these species inhabit open and xeric habitats, as other species within the subfamily Nothurinae [38]. Elegant crested tinamous mostly occupy arid scrub, and avoid thick brushlands as well as tall, dense grasslands [56]. Brushland tinamous prefer semi-open habitat, and are common in semi-open or dense thorn woods, and thorn scrubs [57]. Nothura tinamous occupy semiarid grasslands, savannas and open brushy country in central and western Argentina [44, 55]. In our study region, the occurrence of the three species were determined by the seasonality in precipitation or temperature, the vegetation index (i.e., EVI), and the interaction between seasonality and EVI. Climate seasonality and EVI are good surrogates of plant productivity and biomass [65, 66], that tend to decrease from the northeast towards the southwest in central Argentina, thus generating a plant productivity gradient. Closely related bird species usually show some degree of spatial segregation along plant productivity gradients [20, 23, 27]. Elegant crested tinamous occupied the less productive and more open habitats, mainly those located in the west and south, while brushland and nothura tinamous occurred in more productive and closer habitats. The occurrence of each species across this plant productivity gradient thus agrees with habitat preferences described for these species at broader spatial scales [44, 55–57]. The occurrence of elegant crested tinamous also showed a positive relationship with shrublands and sites with greater cattle activity, which likely indicates the sparser vegetation where this species preferably occurs [67].

The presence of predators, or greater use of preferred sites, could reduce the likelihood of prey occurrence, or alter prey co-occurrence patterns according to species-specific responses to increased predation risk [28, 30]. In the caldén woodland region, tinamou species share similar predators [50]. We found that the distribution of each tinamou species could be related to the presence of one or two predator species. Elegant crested tinamous avoided sites used by pampas cats, while brushland and nothura tinamous showed a positive relationship with sites used by Geoffroy’s cats. Moreover, brushland tinamous preferred sites used by skunks. These results would suggest a negative effect of predation by cats on elegant crested tinamous, positive co-occurrences between Geoffroy’s cats and brushland or nothura tinamous, and a similar positive effect between skunks and brushland tinamous. Because tinamou species are a relatively important prey for Geoffroy’s cats and skunks (opportunistic egg predators) [57, 68], positive co-occurrences between predators and both tinamou species could indicate that sites more likely occupied by tinamous support higher numbers of these potential predators. Therefore, predator abundance seems to have some impact on habitat use of the three tinamou species [20, 69].

However, including habitat use by predators as covariates in the models makes it difficult to assess their role as biotic interactors or rather as indicators of local environmental conditions [70]. We used available information on land cover categories, not quantitative structural variables, as habitat predictors. These categorical variables were relatively unimportant, particularly in the models for brushland and nothura tinamous. Therefore, an alternative, although non-mutually exclusive, explanation is that predator species selected in our best-supported occupancy models could act somewhat as surrogates of missing habitat characteristics [71, 72]. For example, pampas cats are not abundant in semiarid caldén woodlands and preferably occupies grassland habitats, which are mainly sand grasslands in this region [50, 73]. Elegant crested tinamous avoid these grasslands [56], and the occurrence of pampas cats could also be a proxy of habitat variables. Likewise, Geoffroy’s cats are more associated with areas of denser cover in these predominately open habitats [50, 68], which are the sites preferably used by nothura and particularly brushland tinamous [41]. On the other hand, the positive relationship between sites used by brushland tinamous and the activity of skunks could indicate unmeasured habitat variables, such as less human-disturbed areas [50, 74], or sites with greater availability of invertebrates [74], that make up a large portion of brushland tinamous’ diet [43, 57].

### Spatial co-occurrence

Models including species interactions, and simultaneously controlling for environmental requirements of each species, suggested that biotic interactions among tinamous were relatively unimportant in this region. We found a positive association between brushland and nothura tinamous, which could be the result of their overlapping habitat use [36]. Although nothura tinamous were scarce in the caldén woodland region, coexistence in sites where brushland and nothura tinamous co-occur could be enabled by resource partitioning. Brushland tinamous consume more insects and other invertebrates than do nothura tinamous, which include more plant material in its diet [43, 44, 55, 57]. On the other hand, elegant crested and nothura tinamous tended to be negatively associated, likely due to differences in their habitat requirements. Moreover, elegant crested tinamous consume proportionally more seeds and other plant material than do nothura tinamous [43, 44, 55, 56], suggesting that these species also show some degree of resource partitioning where they coexist. Coexistence among ecologically similar species usually involves several niche dimensions, including the dietary component of the niche [19, 20], and it is likely that diet differences further facilitate niche segregation among our studied tinamou species.

### Temporal partitioning

All tinamou species showed similar diurnal activity with two distinct peaks of activity throughout the day. This pattern of diurnal activity is common in tinamou species and other ground-dwelling birds, with variations in activity levels during the day depending on habitat or season [20, 24, 48, 75]. We found wide overlap in overall diel activity patterns among the three tinamous. These general patterns of temporal activity are presumably a response to common abiotic conditions or other biotic variables, such as fluctuations in food availability or predation risk (e.g., potential predators are mainly nocturnal and crepuscular in this region [50]) [27, 76]. However, when comparing the activity times of subordinate tinamous between sites with higher and lower occupancy of the potentially dominant elegant crested tinamous, subordinate tinamous appear to shift their activity patterns to reduce encounters with elegant crested tinamous. These results suggest some degree of temporal partitioning to reduce interspecific competition (although sample size was low in some cases), which is consistent with previous research on temporal segregation in similar sympatric species of different taxa [77, 78].

## Conclusion

Species can modify one or several niche dimensions, such us space use, diel activity, or resource consumption, in response to competition [18, 20]. Our results indicate that habitat use appear to be the main factor determining the distribution and areas of co-occurrence in sympatric tinamou species in the caldén woodland region of central Argentina. Therefore, segregation along the habitat gradient seem to reduce competition among these tinamou species [20, 23, 24, 27]. Patterns of habitat use showed by each tinamou species might have arisen in sympatry, although not necessarily due to competition, or evolved in allopatry. On the other hand, differences in the diet of each species can further facilitate their coexistence where they co-occur [20, 36]. Moreover, the three species exhibited similar patterns of overall daily activity, with some evidence suggesting temporal partitioning as a mechanism to reduce competition in areas of coexistence. Understanding the environmental factors determining tinamou distributions and patterns of co-occurrence is necessary to predict species responses to changes in climate conditions or disturbance regimes [11, 79]. Given the limited extent of protected areas in this ecosystem, changes in human activities, such as increasing logging extraction, agriculture and livestock production, or modifying fire regimes [46, 80], could alter tinamou co-occurrence patterns and the strength of competitive interactions [81]. Climate projections suggest a general warming across all Argentina, and similar or a slight reduction in precipitation in the central part of the country, although with higher variation in seasonal rainfall [82, 83]. Warmer and more seasonal scenarios in caldén woodlands of central Argentina could thus benefit elegant crested tinamous over the other two tinamou species. Nevertheless, it is likely that the combined effect of climate and land use changes will negatively impact the three tinamou species. Finally, tinamous are important prey species for carnivores in this ecosystem [50], and are also hunted by humans [55–57]; thus management of tinamou populations has implications for the conservation of wildlife populations and local economy in this region.

## Supporting information

S1 Table Mean, standard deviation, and range values for the climatic, habitat (proportions), anthropogenic (encounter rates), and biotic (encounter rates) continuous variables estimated at every camera trap station (*n* = 706) in the caldén woodland region of central Argentina. (PDF)

S2 Table Top single-species detection models (ΔAIC ≤ 2) used to evaluate the effects of temporal, habitat, anthropic and survey covariates on the probability of detection (*p*), while holding occupancy as constant (*Ψ*(.)), for elegant crested tinamous (*Eudromia elegans*), brushland tinamous (*Nothoprocta cinerascens*), and nothura tinamous (*Nothura* spp.) in caldén woodlands in central Argentina. Variables with greater effect (i.e., 95% confidence intervals do not include zero) are marked in bold. Positive (+) and negative (-) signs denote direction of explanatory variables. Variable names refer to autumn-winter season (autumn-winter), Julian date (date), closed caldén woodlands (closed woodland), open caldén woodlands (open woodland), shrublands (shrubland), encounter rates of humans (human activity), camera stations installed in trails (trail), and camera trapping effort (trapping effort).
(PDF)

S3 Table Estimates, standard errors, and 95% confidence intervals from best single-species occupancy models for the effects of covariates on the probability of occupancy and detection of elegant crested tinamous (*Eudromia elegans*), brushland tinamous (*Nothoprocta cinerascens*), and nothura tinamous (*Nothura* spp.) in the caldén woodland region in central Argentina. Variables in the occupancy part of the models include: temperature seasonality (Tseas), precipitation seasonality (ppseas), enhanced vegetation index (EVI), closed caldén woodlands (closed woodland), open caldén woodlands (open woodland), shrublands (shrubland), and encounter rates of cattle (cattle), Geoffroy’s cat (gcat) and pampas cat (pcat). Variables in the detection part of the models include: autumn-winter season (autumn-winter), Julian date (date), closed caldén woodlands (closed woodland), open caldén woodlands (open woodland), shrublands (shrubland), encounter rates of humans (human activity), camera stations installed in trails (trail), and camera trapping effort (trapping effort).
(PDF)

S4 Table Capture histories and time recordings for the three tinamou species, and climatic, habitat, anthropogenic, and biotic variables estimated at every camera trap station (*n* = 706) in the caldén woodland region of central Argentina. (XLSX)

S1 Fig Probability of occupancy (± SD) for three tinamou species in caldén woodlands in central Argentina. Elegant crested tinamous (*Eudromia elegans*; black), brushland tinamous (*Nothoprocta cinerascens*; grey), and nothura tinamous (*Nothura* spp.; white).
(PDF)

S2 Fig Kernel density estimation on circular data from camera-trapping records of activity patterns of three tinamou species in caldén woodlands of central Argentina. Grey shading indicates the overlap in species activity and is reported as the coefficient of overlap (Δ_4_) with 95% confidence intervals between brackets. Sample sizes in parentheses indicate the number of detections for each species. Vertical black dashed lines represent average sunrise and sunset at the study area.
(PDF)

S3 Fig Seasonal daily activity patterns of three tinamou species based on kernel density estimation on circular data from camera-trapping records in caldén woodlands of central Argentina. Sample sizes indicate the number of detections for each species. Vertical black dashed lines represent average sunrise and sunset at the study area. Grey boxes show the repetition of activity from one day to the next. Rugs indicate occurrences of photos for each species.
(PDF)
