# Supplementary material for: Habitat partitioning among sympatric tinamous in semiarid woodlands of central Argentina
Source: PLoS One. 2024 Jan 19;19(1):e0297053. doi: 10.1371/journal.pone.0297053 (PMC10798496; doi:10.1371/journal.pone.0297053)
Supplement: S1 Table — (PDF) [file pone.0297053.s001.pdf]

S1 Table. Mean, standard deviation, and range values for the climatic, habitat (proportions), anthropogenic (encounter rates), and biotic (encounter rates) continuous variables estimated at every camera trap station (*n* = 706) in the caldén woodland region of central Argentina.

Variables	Mean	SD	Range
Temperature seasonality	58.57	1.20	56.45-61.67
Precipitation seasonality	66.77	4.85	57.32-80.09
Enhanced Vegetation Index	0.18	0.03	0.08-0.30
Closed caldén woodland	0.22	0.39	0.00-1.00
Open caldén woodland	0.18	0.36	0.00-1.00
Shrubland	0.22	0.39	0.00-1.00
Cattle	0.23	0.27	0.00-1.00
Human	0.01	0.03	0.00-0.33
Puma	0.01	0.02	0.00-0.14
Pampas fox	0.15	0.14	0.00-0.79
Geoffroy's cat	0.03	0.07	0.00-0.83
Pampas cat	0.01	0.01	0.00-0.10
Molina's hog-nosed skunk	0.01	0.03	0.00-0.29
Armadillos	0.02	0.04	0.00-0.37
Camera trapping effort (*n* days)	30.68	7.51	4.00-50.00
