# Supplementary material for: Habitat partitioning among sympatric tinamous in semiarid woodlands of central Argentina
Source: PLoS One. 2024 Jan 19;19(1):e0297053. doi: 10.1371/journal.pone.0297053 (PMC10798496; doi:10.1371/journal.pone.0297053)
Supplement: S2 Table — Variables with greater effect (i.e., 95% confidence intervals do not include zero) are marked in bold. Positive (+) and negative (-) signs denote direction of explanatory variables. Variable names refer to autumn-winter season (autumn-winter), Julian date (date), closed caldén woodlands (closed woodland), open caldén woodlands (open woodland), shrublands (shrubland), encounter rates of humans (human activity), camera stations installed in trails (trail), and camera trapping effort (trapping effort). (PDF) [file pone.0297053.s002.pdf]

S2 Table. Top single-species detection models (ΔAIC ≤ 2) used to evaluate the effects of temporal, habitat, anthropic and survey covariates on the probability of detection (*p*), while holding occupancy as constant (*Ψ*(.)), for elegant crested tinamous (*Eudromia elegans*), brushland tinamous (*Nothoprocta cinerascens*), and nothura tinamous (*Nothura* spp.) in caldén woodlands in central Argentina. Variables with greater effect (i.e., 95% confidence intervals do not include zero) are marked in bold. Positive (+) and negative (-) signs denote direction of explanatory variables. Variable names refer to autumn-winter season (autumn-winter), Julian date (date), closed caldén woodlands (closed woodland), open caldén woodlands (open woodland), shrublands (shrubland), encounter rates of humans (human activity), camera stations installed in trails (trail), and camera trapping effort (trapping effort).

Species/Model	AIC	ΔAIC	*W*	*K*
Elegant crested tinamou				
Ψ (.); *p*(+autumn-winter, -closed woodland, **-shrubland**, **-human activity**, **+trail**, **-trapping effort**)	3833.40	0.00	0.47	8
Ψ (.); *p*(**-closed woodland**, **-shrubland**, **-human activity**, **+trail**, **-trapping effort**)	3835.33	1.92	0.18	7
Brushland tinamou				
Ψ (.); *p*(**-autumn-winter**, +closed woodland, +**shrubland**, **+trail**, +trapping effort)	2508.59	0.00	0.31	7
Ψ (.); *p*(**-autumn-winter**, **+closed woodland**, +**shrubland**, -human activity, +trail, **+trapping effort**)	2509.92	1.33	0.16	8
Ψ (.); *p*(**-autumn-winter**, **+closed woodland**, +**shrubland**, **+trapping effort**)	2509.97	1.39	0.16	6
Nothura tinamou				
Ψ (.); *p*(**-date**, **-date^2**, -open woodland, -human activity, +**trapping effort**)	1212.56	0.00	0.17	7
Ψ (.); *p*(**-date**, **-date^2**, -open woodland, -human activity, -trail, +**trapping effort**)	1213.31	0.75	0.12	8
Ψ (.); *p*(**-autumn-winter**, -open woodland, -human activity, +**trapping effort**)	1213.32	0.76	0.12	6
Ψ (.); *p*(**-autumn-winter**, -open woodland, +**trapping effort**)	1213.40	0.84	0.11	5
Ψ (.); *p*(**-date**, **- date^2**, -human activity, +**trapping effort**)	1213.41	0.85	0.11	6

Species/Model	AIC	ΔAIC	*W*	*K*
Ψ (.); *p*(**-autumn-winter**, +**trapping effort**)	1213.54	0.98	0.10	4
Ψ (.); *p*(**-date**, **- date^2**, +**trapping effort**)	1214.19	1.63	0.07	5
Ψ (.); *p*(**-autumn-winter**, -open woodland, -human activity, -trail, +**trapping effort**)	1214.52	1.96	0.06	7
