# Supplementary material for: Habitat partitioning among sympatric tinamous in semiarid woodlands of central Argentina
Source: PLoS One. 2024 Jan 19;19(1):e0297053. doi: 10.1371/journal.pone.0297053 (PMC10798496; doi:10.1371/journal.pone.0297053)
Supplement: S3 Table — Variables in the occupancy part of the models include: temperature seasonality (Tseas), precipitation seasonality (ppseas), enhanced vegetation index (EVI), closed caldén woodlands (closed woodland), open caldén woodlands (open woodland), shrublands (shrubland), and encounter rates of cattle (cattle), Geoffroy’s cat (gcat) and pampas cat (pcat). Variables in the detection part of the models include: autumn-winter season (autumn-winter), Julian date (date), closed caldén woodlands (closed woodland), open caldén woodlands (open woodland), shrublands (shrubland), encounter rates of humans (human activity), camera stations installed in trails (trail), and camera trapping effort (trapping effort). (PDF) [file pone.0297053.s003.pdf]

S3 Table. Estimates, standard errors, and 95% confidence intervals from best single-species occupancy models for the effects of covariates on the probability of occupancy and detection of elegant crested tinamous (*Eudromia elegans*), brushland tinamous (*Nothoprocta cinerascens*), and nothura tinamous (*Nothura* spp.) in the caldén woodland region in central Argentina. Variables in the occupancy part of the models include: temperature seasonality (Tseas), precipitation seasonality (ppseas), enhanced vegetation index (EVI), closed caldén woodlands (closed woodland), open caldén woodlands (open woodland), shrublands (shrubland), and encounter rates of cattle (cattle), Geoffroy's cat (gcat) and pampas cat (pcat). Variables in the detection part of the models include: autumn-winter season (autumn-winter), Julian date (date), closed caldén woodlands (closed woodland), open caldén woodlands (open woodland), shrublands (shrubland), encounter rates of humans (human activity), camera stations installed in trails (trail), and camera trapping effort (trapping effort).

Species/Model	Estimate	SE	95% CI
Elegant crested tinamou			
Occupancy:			
intercept	-0.942	0.134	(-1.199, -0.683)
ppseas	0.888	0.183	(0.599, 1.259)
EVI	-0.268	0.172	(-0.595, 0.066)
ppseas×EVI	0.450	0.142	(0.216, 0.742)
open woodland	0.466	0.105	(0.264, 0.673)
shrubland	0.215	0.116	(-0.005, 0.452)
cattle	0.107	0.107	(-0.104, 0.314)
pcat	-0.370	0.207	(-0.901, -0.051)
Detection:			
intercept	-2.595	0.070	(-2.735, -2.465)
autumn-winter	0.083	0.054	(-0.023, 0.188)
closed woodland	-0.086	0.064	(-0.214, 0.037)
shrubland	-0.222	0.066	(-0.357, -0.098)
human activity	-0.351	0.114	(-0.59, -0.144)
trail	0.185	0.066	(0.058, 0.314)
trapping effort	-0.227	0.068	(-0.363, -0.096)

Species/Model	Estimate	SE	95% CI
Brushland tinamou			
Occupancy:			
intercept	-1.736	0.159	(-2.058, -1.433)
Tseas	-0.309	0.155	(-0.618, -0.012)
EVI	0.917	0.159	(0.618, 1.245)
gcat	0.356	0.184	(0.037, 0.751)
skunk	0.356	0.184	(0.132, 0.630)
Detection:			
intercept	-2.980	0.112	(-3.206, -2.778)
autumn-winter	-0.298	0.093	(-0.482, -0.119)
closed woodland	0.137	0.075	(-0.008, 0.284)
shrubland	0.313	0.086	(0.148, 0.479)
trail	0.162	0.075	(0.016, 0.310)
trapping effort	0.133	0.103	(-0.066, 0.334)
Nothura tinamou			
Occupancy:			
intercept	-2.805	0.325	(-3.474, -2.213)
ppseas	-1.379	0.334	(-1.944, -0.764)
EVI	0.421	0.242	(-0.035, 0.907)
gcat	0.334	0.181	(0.037, 0.762)
Detection:			
intercept	-3.661	0.245	(-4.159, -3.206)
date	-0.679	0.184	(-1.06, -0.332)
date2	-0.385	0.124	(-0.642, -0.152)
open woodland	-0.166	0.138	(-0.45, 0.092)
human activity	-0.381	0.217	(-0.871, 0.015)
trapping effort	0.993	0.325	(0.385, 1.654)
