# Supplementary material for: Habitat partitioning among sympatric tinamous in semiarid woodlands of central Argentina
Source: PLoS One. 2024 Jan 19;19(1):e0297053. doi: 10.1371/journal.pone.0297053 (PMC10798496; doi:10.1371/journal.pone.0297053)
Supplement: S1 Fig — Elegant crested tinamous (Eudromia elegans; black), brushland tinamous (Nothoprocta cinerascens; grey), and nothura tinamous (Nothura spp.; white). (PDF) [file pone.0297053.s005.pdf]

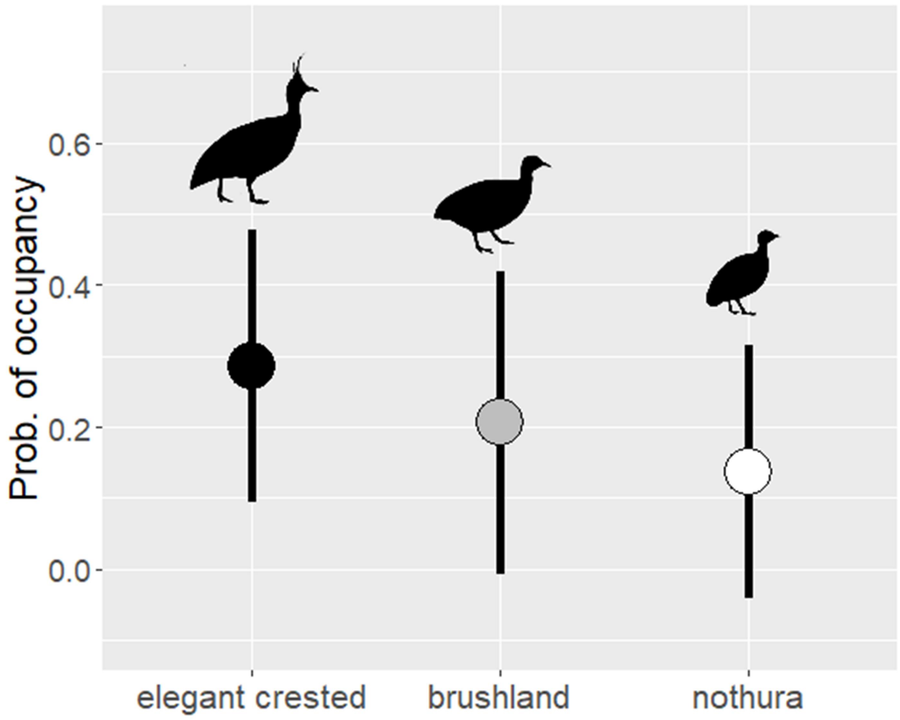

S1 Fig. Probability of occupancy (± SD) for three tinamou species in caldén woodlands in central Argentina. Elegant crested tinamous (*Eudromia elegans*; black), brushland tinamous (*Nothoprocta cinerascens*; grey), and nothura tinamous (*Nothura* spp.; white).
