# Supplementary material for: Habitat partitioning among sympatric tinamous in semiarid woodlands of central Argentina
Source: PLoS One. 2024 Jan 19;19(1):e0297053. doi: 10.1371/journal.pone.0297053 (PMC10798496; doi:10.1371/journal.pone.0297053)
Supplement: S2 Fig — Grey shading indicates the overlap in species activity and is reported as the coefficient of overlap (Δ4) with 95% confidence intervals between brackets. Sample sizes in parentheses indicate the number of detections for each species. Vertical black dashed lines represent average sunrise and sunset at the study area. (PDF) [file pone.0297053.s006.pdf]

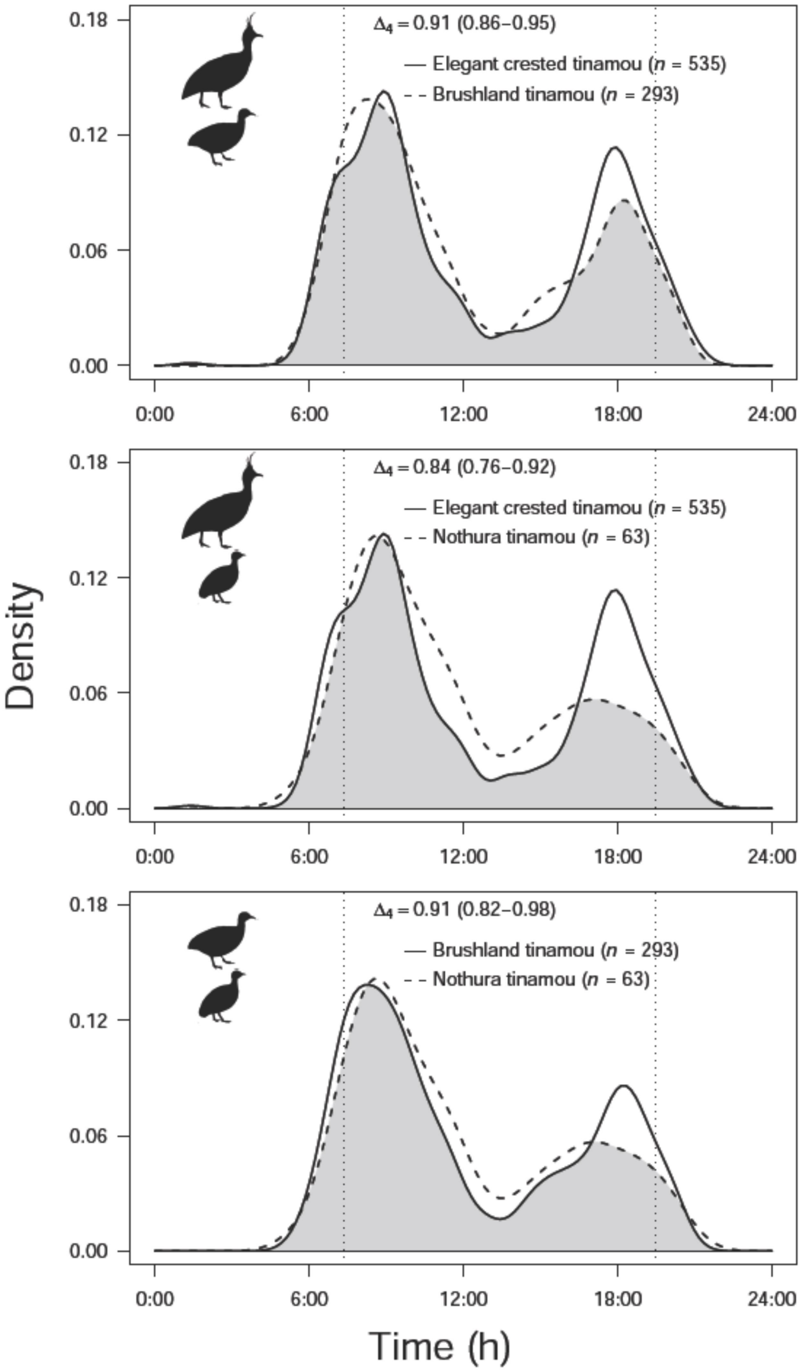

S2 Fig. Kernel density estimation on circular data from camera-trapping records of activity patterns of three tinamou species in caldén woodlands of central Argentina. Grey shading indicates the overlap in species activity and is reported as the coefficient of overlap (Δ_4) with 95% confidence intervals between brackets. Sample sizes in parentheses indicate the number of detections for each species. Vertical black dashed lines represent average sunrise and sunset at the study area.
