# Supplementary material for: Habitat partitioning among sympatric tinamous in semiarid woodlands of central Argentina
Source: PLoS One. 2024 Jan 19;19(1):e0297053. doi: 10.1371/journal.pone.0297053 (PMC10798496; doi:10.1371/journal.pone.0297053)
Supplement: S3 Fig — Sample sizes indicate the number of detections for each species. Vertical black dashed lines represent average sunrise and sunset at the study area. Grey boxes show the repetition of activity from one day to the next. Rugs indicate occurrences of photos for each species. (PDF) [file pone.0297053.s007.pdf]

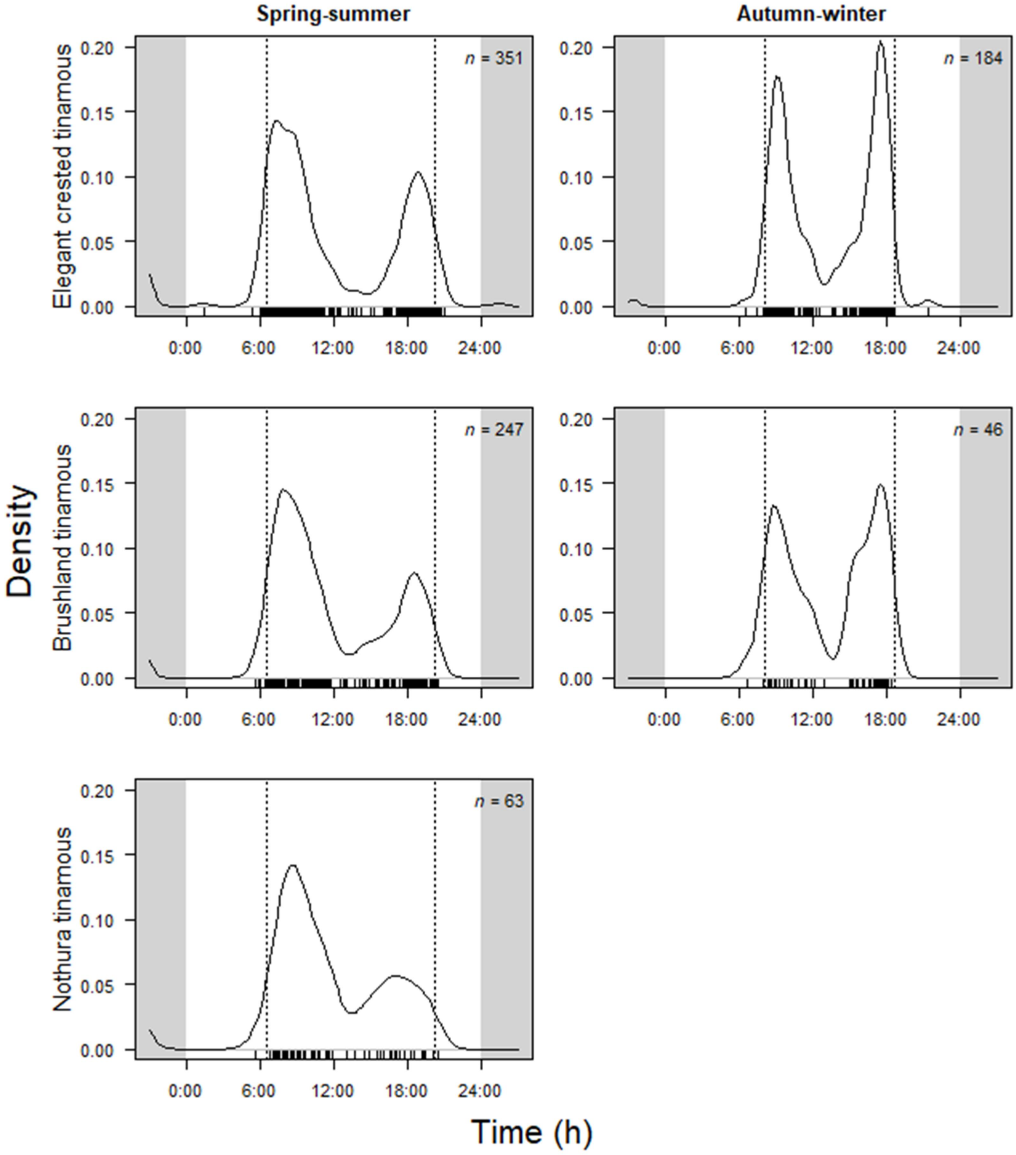

S2 Fig. Seasonal daily activity patterns of three tinamou species based on kernel density estimation on circular data from camera-trapping records in caldén woodlands of central Argentina. Sample sizes indicate the number of detections for each species. Vertical black dashed lines represent average sunrise and sunset at the study area. Grey boxes show the repetition of activity from one day to the next. Rugs indicate occurrences of photos for each species.
